# Leucine degradation metabolite ratio as a measure of meropenem susceptibility in carbapenem-resistant *Klebsiella pneumoniae*

**DOI:** 10.1128/spectrum.00099-26

**Published:** 2026-06-10

**Authors:** Breanna Dixon, Kamila Schmidt, Stephen J. Fowler, Drupad K. Trivedi, Tim Felton, Waqar M. Ahmed

**Affiliations:** 1 Division of Immunology, Immunity to Infection and Respiratory Medicine, School of Biological Sciences, Faculty of Biology, Medicine and Health, University of Manchester 5292, Manchester, United Kingdom; 2 Manchester Institute of Biotechnology, Department of Chemistry, University of Manchester 5292, Manchester, United Kingdom; 3 NIHR-Manchester Biomedical Research Centre, Manchester University Hospitals NHS Foundation Trust 5293 , Manchester, United Kingdom; Rowan University Cooper Medical School, Camden, New Jersey, USA

**Keywords:** metabolomics, *Klebsiella pneumoniae*, antibiotic susceptibility testing, GC-MS, volatile organic compounds, rapid diagnostics, antibiotic resistance, meropenem, VOC, leucine, carbapenem resistance

## Abstract

Rapid detection of carbapenem-resistant *Klebsiella pneumoniae* (CRKP) is essential for effective clinical management and surveillance. This study investigated volatile organic compounds (VOCs) as phenotypic biomarkers of carbapenem resistance, with the goal of developing a translationally viable diagnostic metric. Sixteen clinical *K. pneumoniae* isolates comprising both CRKP and carbapenem-susceptible *K. pneumoniae* (CSKP) strains were cultured for 6 h in headspace vials, with and without meropenem, and VOCs were extracted using polydimethylsiloxane-coated probes. Volatiles were analyzed by thermal desorption-gas chromatography-mass spectrometry. Under meropenem stress, CRKP and CSKP exhibited distinct VOC profiles, with two leucine-derived metabolites, 3-methyl-1-butanol (syn. isoamyl alcohol) and 3-methylbutanal (syn. isovaleraldehyde), emerging as key discriminatory features. The ratio of these two metabolites accurately classified resistance phenotype, achieving 100% sensitivity and 94.1% specificity. The ratio also correlated significantly with minimum inhibitory concentration and zone of inhibition values. Stable isotope tracing confirmed their origin from leucine catabolism, and metabolic supplementation experiments suggested a functional role for 3-methyl-1-butanol in promoting growth under antibiotic stress. These findings show that antimicrobial resistance is accompanied by detectable shifts in volatile metabolism and that VOCs can serve as reliable indicators of resistance phenotype. The 3-methyl-1-butanol/3-methylbutanal ratio presents a robust biomarker of meropenem resistance, detectable within 6 h of culture growth, and supports the further development of volatilome-based diagnostics for clinical microbiology.

**IMPORTANCE:**

Carbapenem-resistant *Klebsiella pneumoniae* pose a major threat to patient outcomes and hospital infection control, yet current diagnostic methods for carbapenem resistance are time-consuming or require specific molecular testing. This study demonstrates that meropenem resistance is associated with reproducible and quantifiable changes in volatile metabolism that can be detected within 6 h of culture. We identify a simple ratio of two leucine-derived volatile organic compounds, 3-methyl-1-butanol and 3-methylbutanal, that discriminates resistant from susceptible *K. pneumoniae* strains and correlates with conventional phenotypic susceptibility measures. Importantly, this ratio reflects a functional metabolic response to antibiotic stress rather than the presence of specific resistance genes, supporting its broad applicability across resistance mechanisms. These findings establish volatile metabolites as rapid, phenotypic indicators of carbapenem resistance and provide a foundation for developing volatilome-based diagnostics that could accelerate antimicrobial susceptibility testing in routine clinical microbiology laboratories.

## INTRODUCTION

*Klebsiella pneumoniae* is an opportunistic pathogen frequently implicated in severe nosocomial infections, including pneumonia, bacteremia, and urinary sepsis (1). Carbapenem-resistant *K. pneumoniae* (CRKP) represents a significant threat to global public health, contributing to the rising challenge of antibiotic resistance in healthcare settings (2). The emergence of CRKP has led to a reduction in treatment options, resulting in higher morbidity, mortality, and healthcare costs (3–5). Rapid and accurate detection of CRKP is therefore essential for effective infection control and timely therapeutic intervention (6).

Current diagnostic approaches for CRKP primarily rely on genomic and culture-based methods, including broth microdilution, disk diffusion susceptibility testing, and the modified carbapenem inactivation method. While these techniques offer high specificity, they are limited by sensitivity issues, long test turnaround times, and/or high reagent costs per test (7–9). Furthermore, targeted molecular methods such as PCR may fail to detect novel resistance genes or mechanisms, particularly those not directly linked to protein synthesis (10). Importantly, genotypic data do not always correlate with phenotypic resistance, making it an unreliable predictor of antibiotic efficacy without accompanying susceptibility testing (11, 12). Such limitations can delay appropriate treatment, resulting in poorer patient outcomes and increased infection transmission within healthcare settings. Consequently, there is a need for novel diagnostic strategies that enable faster detection of carbapenem resistance.

Microorganisms, including antibiotic-resistant bacterial strains, produce a diverse array of volatile organic compounds (VOCs) as part of their metabolic processes. Studies employing gas chromatography-mass spectrometry have demonstrated that bacterial VOC profiles are species specific, highlighting their potential for microbial identification (13–17). Certain VOCs are already exploited in clinical biochemical assays; for example, the indole test differentiates bacteria based on their ability to produce the enzyme tryptophanase, which converts tryptophan into indole. Building on this, microbial VOCs have been proposed as rapid, non-invasive biomarkers for detecting infectious diseases (18–21). Notably, VOC-based assays can be applied to biological sample matrices, including exhaled breath, feces, and urine, allowing real-time monitoring of microbial activity without disrupting the host or the culture environment. More recently, metabolomic approaches have exploited VOCs for the assessment of antibiotic susceptibility and discrimination of antibiotic-resistant bacteria (22–27).

In this study, we present a method for the detection of CRKP using specific volatile microbial metabolites. Using thermal desorption-gas chromatography-mass spectrometry (TD-GC-MS), we identify VOC signatures associated with carbapenem resistance in *K. pneumoniae* and propose a diagnostic metric, enabling the classification of CRKP and *c*arbapenem-susceptible *K. pneumoniae* (CSKP) and determination of minimum inhibitory concentration (MIC). Furthermore, we confirm the involvement of the leucine degradation pathway in driving this altered metabolic phenotype using deuterated leucine supplementation. Our findings demonstrate the potential of VOC-based diagnostics as a tool for the early detection of antibiotic-resistant pathogens, which may be integrated into clinical workflows.

## MATERIALS AND METHODS

### Bacterial isolates

A total of 16 pathogenic *Klebsiella pneumoniae* isolates were used in the study (CSKP; *n* = 7, CRKP; *n* = 9). All strains were clinical isolates sourced from the North Bristol NHS Trust and Manchester University NHS Foundation Trust, except for five clinical reference strains obtained from the National Collection of Type Cultures (UK Health Security Agency, UK): NCTC 13438, NCTC 13442, NCTC 13443, ATCC 13883, and ATCC 13887. Representative carbapenem-susceptible and carbapenem-resistant *K. pneumoniae* isolates were selected for individual experiments to ensure that the observed metabolic responses were reproducible across independent strains and not restricted to a single isolate pair.

### Conventional antibiotic susceptibility testing

Susceptibility testing was performed according to the European Committee on Antimicrobial Susceptibility Testing (EUCAST) guidelines using the Kirby-Bauer method with 10 µg meropenem disks (Oxoid, UK) (28). Zone of inhibition (ZOI) diameters were recorded after 18 h incubation at 35°C. Three independent experiments of each assessment were performed. Results were validated using the American Type Culture Collection (ATCC) quality control strain *E. coli* ATCC 25922. Measurements were compared to EUCAST breakpoints to characterize strains as “*susceptible*,” “*susceptible*, *increased exposure*,” or “*resistant*” (29).

The broth microdilution method was implemented for the determination of the meropenem MIC (30). Each strain was assayed in triplicate across a range of 0.004–128 µg mL^−1^ meropenem (meropenem trihydrate; APExBIO Technology, USA). The method was performed three times across different days. Results were interpreted using the 2022 EUCAST breakpoint criteria (29).

Susceptibility results can be found in Table S1.

### Headspace sampling of bacterial cultures with meropenem disks

For each of the 16 isolates, a 1 µL loop of overnight culture was inoculated in 2 mL tryptic soy broth (TSB) within a 10 mL headspace vial containing a 10 µg meropenem disk (*n* = 3). Meropenem-free setups were prepared in parallel (*n* = 3). Vials were crimp sealed, and high-capacity sorptive extraction probes (HiSorb, Markes International, UK) with a polydimethylsiloxane sorbent coating were inserted into the headspace through the vial septa. Headspace was passively collected over a 6 h incubation period at 37°C with 180 rpm agitation, after which the probes were removed and inserted into cleaned empty stainless steel TD tubes for analysis.

### Meropenem susceptibility testing using VOC analysis

Overnight cultures of four isolates (KP007, KP019, KP025, and KP030) were diluted to 0.1 OD_600_ in TSB, and 100 µL of each dilution was combined with 900 µL meropenem-supplemented TSB media within a 10 mL headspace vial. Isolates were selected based on their MIC value. Three levels of meropenem concentration were prepared for each isolate: 0 µg mL^−1^, 1 µg mL^−1^, and 16 µg mL^−1^. Three replicates of each isolate were grown in each concentration. Headspace was passively collected using HiSorb probes over a 6 h incubation period at 37°C with 180 rpm agitation.

### Specificity testing of diagnostic metric

For specificity testing, isolates KP003, KP009, KP031, and KP034 were treated with 10 µg gentamicin and 5 µg ciprofloxacin disks. Headspace analysis was performed as before. Additionally, susceptibility to each antibiotic was recorded for each isolate using the Kirby-Bauer disk diffusion method (Table S2).

### Supplementation with 3-methylbutanal and 3-methyl-1-butanol

On the basis of our initial results, microdilution assays were performed to evaluate the effects of 3-methylbutanal (syn. isovaleraldehyde) or 3-methyl-1-butanol (syn.isoamyl alcohol) on bacterial growth. A twofold serial dilution of 3-methylbutanal (Sigma Aldrich, UK) or 3-methyl-1-butanol (Sigma Aldrich, UK) was prepared in TSB, ranging from 0 mM to 32 mM. Meropenem was tested at concentrations of 0 and 0.032 µg mL^−1^ for isolate KP001 (CSKP) and 0 and 16 µg mL^−1^ for isolate KP008 (CRKP) in combination with 3-methyl-1-butanol.

Bacterial cultures were adjusted to an OD_600_ of 0.001 and inoculated into each well of a 96-well plate. The plates were incubated at 37°C for 18 h, and bacterial growth was assessed by measuring OD_600_ using a microplate reader (BMG Clariostar Multimode Plate Reader, Germany) at 15 min intervals. Four biological replicates were performed for each setup.

### d10-Leucine supplementation

TSB was supplemented with d10-leucine (Sigma Aldrich, UK) at a concentration equivalent to its native leucine content (1.43 mg mL^−1^). Two *K. pneumoniae* isolates, KP003 (CSKP) and KP007 (CRKP), were grown in TSB with and without d10-leucine. Headspace sampling of bacterial cultures exposed to 10 µg meropenem disks was conducted as described earlier.

### Data acquisition

Probes were dry purged with N_2_ at 50 mL min^−1^ for 4 min to remove water residue then analyzed by TD-GC-MS. Samples were randomized to counteract sampling batch effects, and a gaseous internal standard (1 ppmV p-bromofluorobenzene in N_2_; Thames Restek, UK) was spiked onto each sample prior to desorption. VOCs were thermally desorbed at 280°C for 10 min (TD100, Markes International, UK) then transferred to a cryofocusing trap maintained at 0°C, which was flash heated to 300°C for 2 min. Samples were injected into the GC with a 1:10 split ratio. VOC separation was performed on an Agilent 7890B GC (Agilent Technologies, UK) using an Agilent DB-5ms column (30 m × 0.25 mm × 0.25 μm) with constant helium flow (1 mL min^−1^). The GC column oven was set to a linear temperature ramp program with an initial temperature of 35°C, increasing at 7.5°C min^−1^ to 260°C. After GC separation, VOCs were transferred to an Agilent 7010 MS with EI+ source set to 70 eV and 230°C, and a triple quadrupole mass analyzer in full scan mode across a range of 40–300 *m/z* with an acquisition rate of 5 Hz.

### Data preprocessing and analysis

Spectral deconvolution was performed using MassHunter Quantitative Analysis software (Agilent Technologies, UK) with a retention window size factor of 100 and delta *m/z* tolerance of 0.3 AMU left/0.7 AMU right. Detected peaks were aligned using a retention time window of ±0.1 min, and tentative peak identifications were performed by comparing mass spectra against the NIST v23 mass spectral library. Only compounds with a mass spectral match factor ≥75% were selected, with annotations listed as the compound name of the top match score.

All subsequent data analysis was performed in R (v4.3.1). Integrated peak areas were normalized against the p-bromofluorobenzene internal standard. Peaks resulting from suspected environmental contaminants and artifacts were removed from analysis, e.g., siloxanes and phthalate-derived compounds. For missing values, a nominal value of 1/5 of the minimum positive value was assigned to each variable. For statistical analysis, data were log transformed and autoscaled with package “*mdatools*” (31). Univariate analysis was performed using two-way *t*-tests. The package “*Hmisc*” was used for correlation analysis with Pearson method, and “*pROC*” was used for ROC analysis (32, 33). Mean feature ratios and corresponding standard errors were calculated from individual replicate data.

## RESULTS

### Carbapenem-resistant *K. pneumoniae* exhibit distinct VOC profile

We developed a simple method that could serve as a means of discerning carbapenem resistance, which is faster than conventional methods. Using the classical carbapenem-inactivation method as a basis, we inoculated isolates in TSB media with and without a 10 µg meropenem disk and measured the headspace volatiles produced after 6 h. In doing so, we eliminated the need for the usual 18–24 h incubation step, which follows the initial 6 h step with the meropenem disk.

We performed headspace VOC profiling of 16 pathogenic *K. pneumoniae* isolates using TD-GC-MS, and a total of 80 peaks were observed across all samples after removal of artifact peaks. Seven VOCs differed significantly (*P* ≤ 0.05) between the CRKP and CSKP groups within the untreated condition, while 20 VOCs were observed to be significantly different in the treated condition.

**Fig 1:**
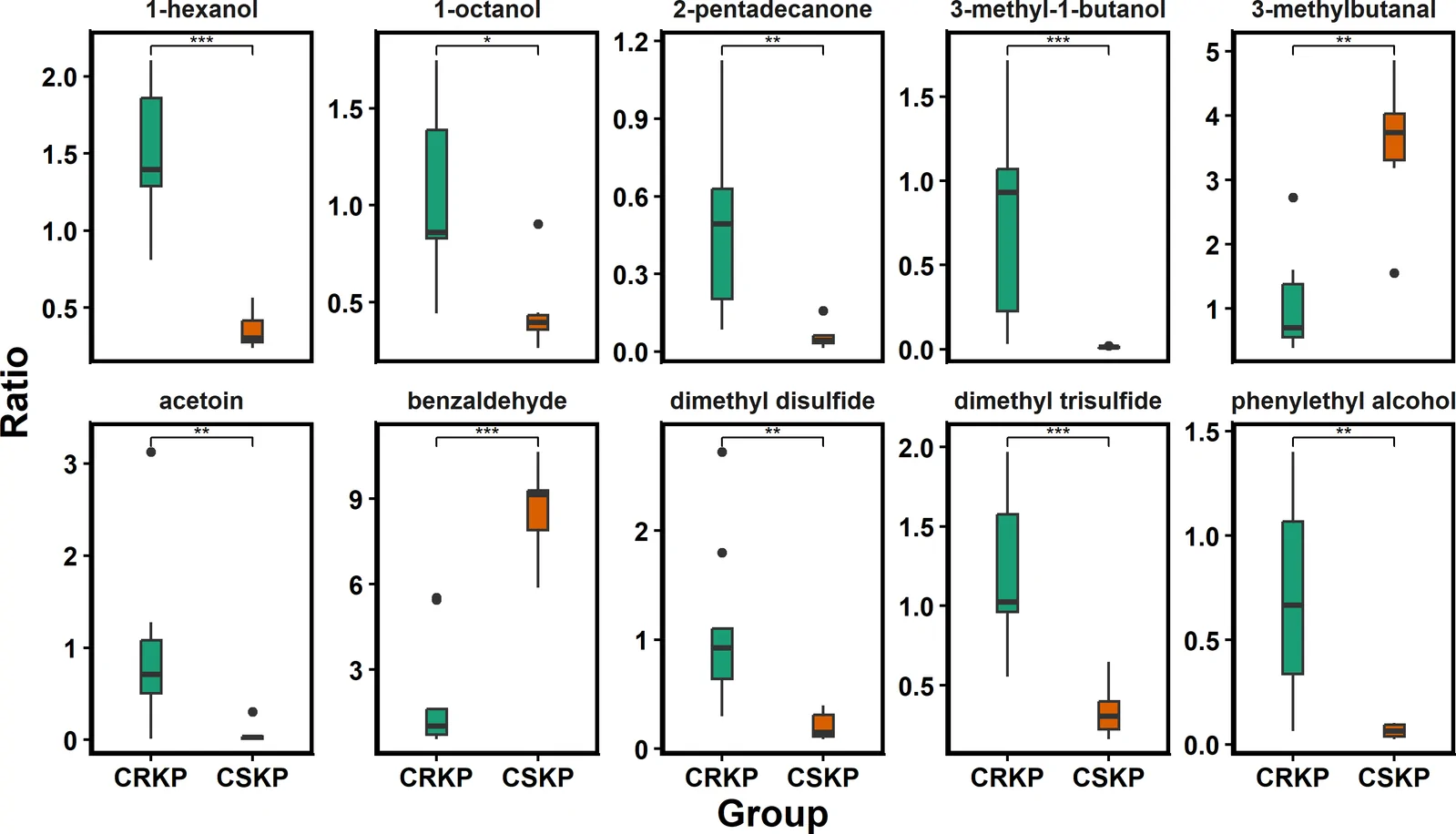
Mean treated-to-untreated ratios of the 10 significantly differential VOCs between CRKP and CSKP groups. R, CRKP; S, CSKP. ***P ≤ 0.001, **P ≤ 0.01, and *P ≤ 0.05.

The mean ratio of peak intensity in the treated condition to untreated condition was calculated and compared for each individual VOC to identify compounds that were differential between the treatment conditions. A ratio close to 1 suggests that the isolate grew in the presence of meropenem in a similar manner as without meropenem. We identified 10 biologically relevant compounds with significantly different (*P* ≤ 0.05) mean ratios between CRKP and CSKP groups (Fig. 1).

Notably, benzaldehyde and 3-methylbutanal showed an inverse relationship compared to the other compounds, with significantly higher ratios seen in CSKP compared to the CRKP group. Where the mean ratio of treated to untreated peak intensity was 0.97 (SE = 0.07) for the other eight compounds in CRKP, the mean ratios for benzaldehyde and 3-methylbutanal in CSKP were 8.61 (SE = 0.68) and 3.53 (SE = 0.46), respectively. The identities of 3-methylbutanal and 3-methyl-1-butanol were confirmed using authentic chemical standards.

### Use of volatiles for the detection of carbapenem resistance

**Fig 2:**
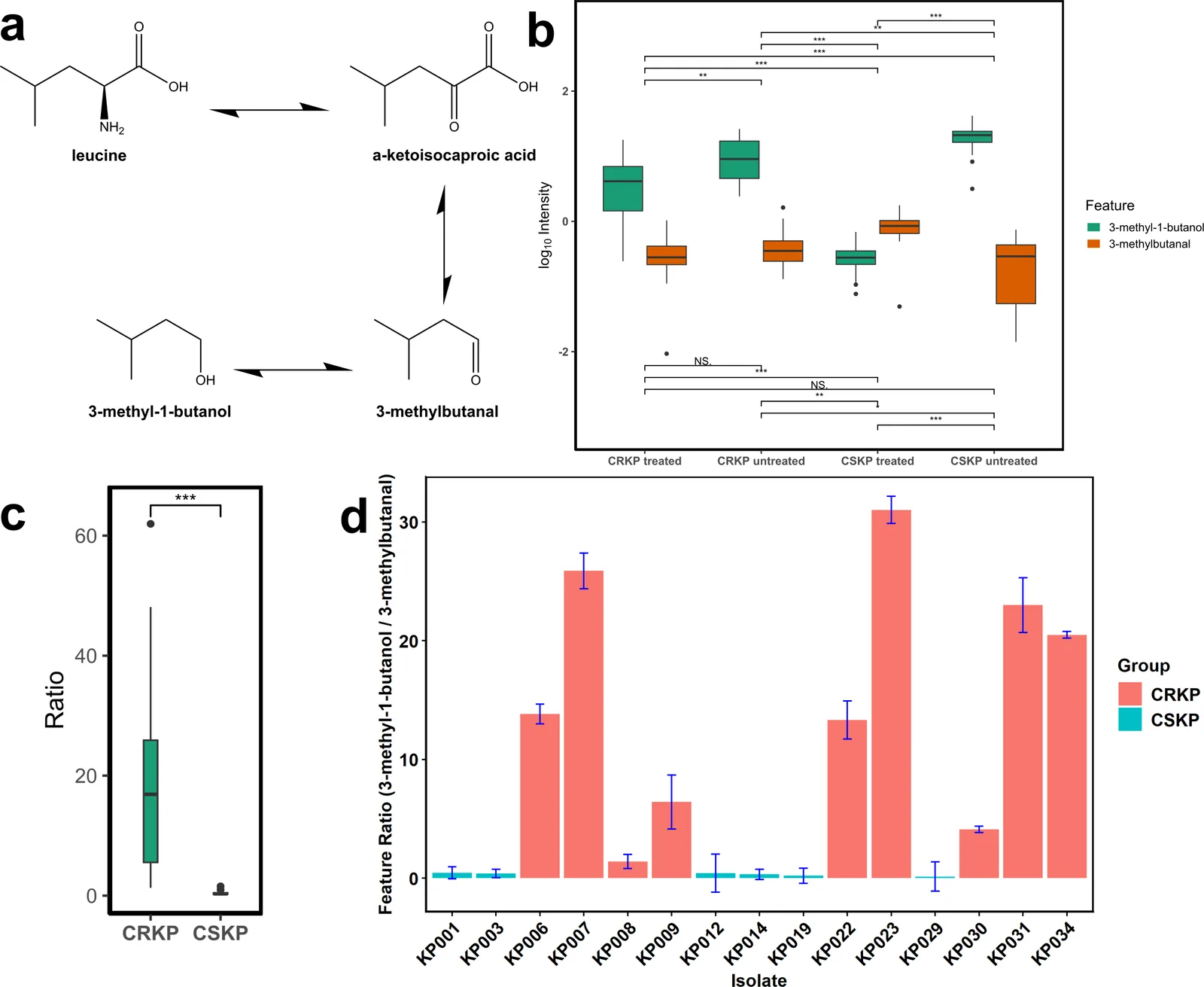
**(a**) Simplified reaction schematic of the leucine degradation pathway. Normalized intensity plots of (**b**) 3-methyl-1-butanol and 3-methylbutanal across each treatment group to allow visualization of condition-influencing ratio direction, (**c**) mean 3-methyl-1-butanol-to-3-methylbutanal ratios of CRKP vs CSKP groups, and (**d**) mean 3-methyl-1-butanol-to-3-methylbutanal ratios of individual *K. pneumoniae* isolates (significance annotations: top, 3-methyl-1-butanol; bottom, 3-methylbutanal). ns, not significant; ***P ≤ 0.001, **P ≤ 0.01, and *P ≤ 0.05.

We then considered the relationship between 3-methylbutanal and 3-methyl-1-butanol, due to their metabolic activity in the leucine degradation pathway (Fig. 2a). The ratio of 3-methyl-1-butanol to 3-methylbutanal was significantly higher in CRKP than CSKP in the treated condition only (*P*; treated = 0.001, untreated = 0.120). In the treated condition, the mean ratio of the CSKP group was 0.438 (SE = 0.09), while the mean ratio for the CRKP group was 18.35 (SE = 2.95), approximately 42 times higher (Fig. 2c). This was largely attributable to the near absence of 3-methyl-1-butanol and elevation of 3-methylbutanal in the treated CSKP group compared to the other sample groups (Fig. 2b).

At the level of the individual isolate, all CRKP isolates demonstrated mean ratios greater than 1.00 between the two compounds (Fig. 2D). To confirm this threshold, we performed receiver operating characteristic (ROC) analysis to determine the optimal threshold value for classification based on Youden’s *J* statistic. This returned a cutoff value of 1.15, with a sensitivity of 100%, a specificity of 94.1%, and an area under the ROC curve of 0.993.

The mean ratios originating from isolates KP008 and KP030 were reduced compared to the other CRKP isolates, and we observed that these two isolates possessed lower MIC values (MIC; KP008 = 32 µg mL^−1^, KP030 = 16 µg mL^−1^) than the other resistant isolates. We performed Pearson’s correlation analysis between the mean 3-methyl-1-butanol to 3-methylbutanal ratio values and the MIC and ZOI values recorded for each isolate during antibiotic susceptibility testing. Strong correlation between the mean ratio and MIC value (*r* = 0.857, *P* = 4.50 × 10^−5^), as well as mean ratio and ZOI (*r* = −0.764, *P* = 8.99 × 10^−4^) was observed. Furthermore, the latter correlation coefficient was not dissimilar to that observed between MIC and ZOI (*r* = −0.739, *P* = 1.66 × 10^−3^).

### 3-methyl-1-butanol/3-methylbutanal ratio as an indicator of meropenem susceptibility

To further investigate the relationship between meropenem susceptibility and the ratio of the compounds 3-methyl-1-butanol and 3-methylbutanal, we grew several strains of varying susceptibilities in increasingly higher concentrations of meropenem (0 µg mL^−1^, 1 µg mL^−1^, and 16 µg mL^−1^). These values represent a control condition, as well as meropenem levels both above and below the conventional MIC breakpoint criteria for susceptibility testing (S ≤ 2 µg mL^−1^ and R > 8 µg mL^−1^).

**Fig 3:**
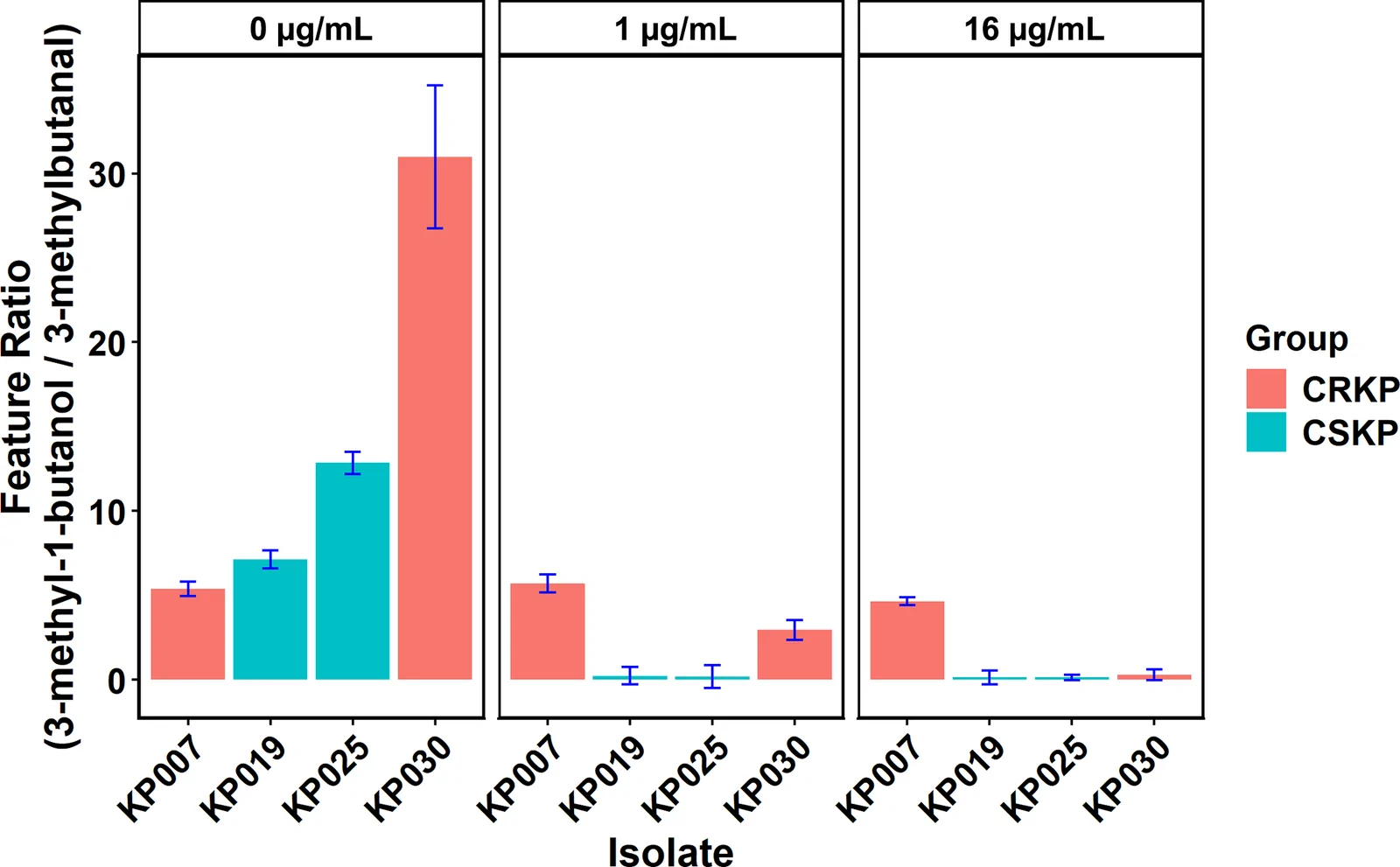
Mean 3-methyl-1-butanol-to-3-methylbutanal ratios of *K. pneumoniae* isolates KP007, KP019, KP025, and KP030 subjected to meropenem-free conditions, as well as meropenem concentrations below (1 µg mL^−1^) and above (16 µg mL^−1^) the clinical breakpoints of meropenem resistance. The MIC values of each isolate were 2, 0.125, 16, and >128 µg mL^−1^, respectively.

Using the mean ratio of these VOCs, we were able to differentiate between isolates with MIC values of ≤2 µg mL^−1^, 16 µg mL^−1^, and >128 µg mL^−1^ (Fig. 3). Susceptibility to a particular concentration of meropenem was demonstrated by the 3-methyl-1-butanol/3-methylbutanal ratio dropping below 1.00. While isolate KP019 demonstrated susceptibility at 1 µg mL^−1^ despite an MIC of 2 µg mL^−1^, this discrepancy was within one doubling dilution, which is generally accepted in clinical practice. The ratios for each of the other isolates were in keeping with their MIC values (KP025 = 0.032 µg mL^−1^, KP030 = 16 µg mL^−1^, and KP007 > 128 µg mL^−1^).

### 3-methyl-1-butanol supplementation shows differential growth in presence of meropenem

To determine whether it was possible to metabolically reprogram and alter the susceptibility of *K. pneumoniae*, we supplemented a susceptible isolate—KP001 (MIC: 0.064 µg mL^−1^)—and a resistant isolate—KP008 (MIC: 32 µg mL^−1^)—with 3-methyl-1-butanol and challenged them with a sub-MIC level of meropenem.

**Fig 4:**
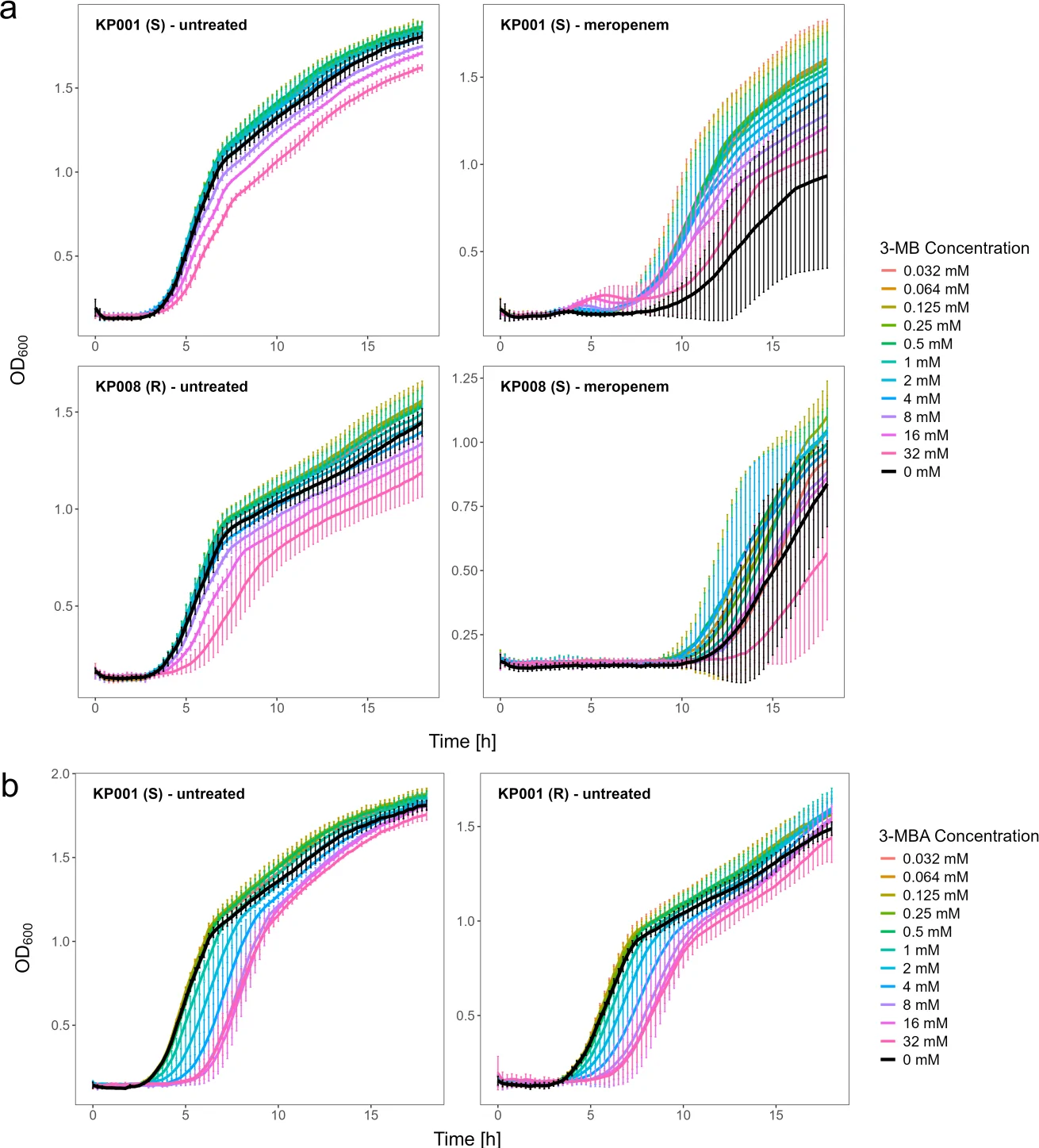
KP001 and KP008 grown in varying concentrations of (**a**) 3-methyl-1-butanol (3-MB) in antibiotic-free and sub-MIC meropenem (KP001; 0.032 µg mL^−1^, KP008; 16 µg mL^−1^) conditions and (**b**) 3-methylbutanal (3-MBA). Growth curves represent the mean values of four replicates with SD plotted as error bars.

For both isolates, 3-methyl-1-butanol supplementation increased growth rates at concentrations below 4 mM in the absence of meropenem, whereas higher concentrations inhibited growth (Fig. 4a). Under sub-MIC meropenem conditions, 3-methyl-1-butanol completely mitigated the growth reduction seen in its absence, with all supplemented concentrations except KP008 at 32 mM showing increased growth rates and maximum growth compared to the control. Supplementation with 3-methylbutanal in antibiotic-free conditions (Fig. 4b) revealed an inhibitory effect on growth at concentrations of 1 mM and above. Concentrations lower than this exhibited increased growth compared to the control.

### Involvement of leucine degradation metabolic pathway

**Fig 5:**
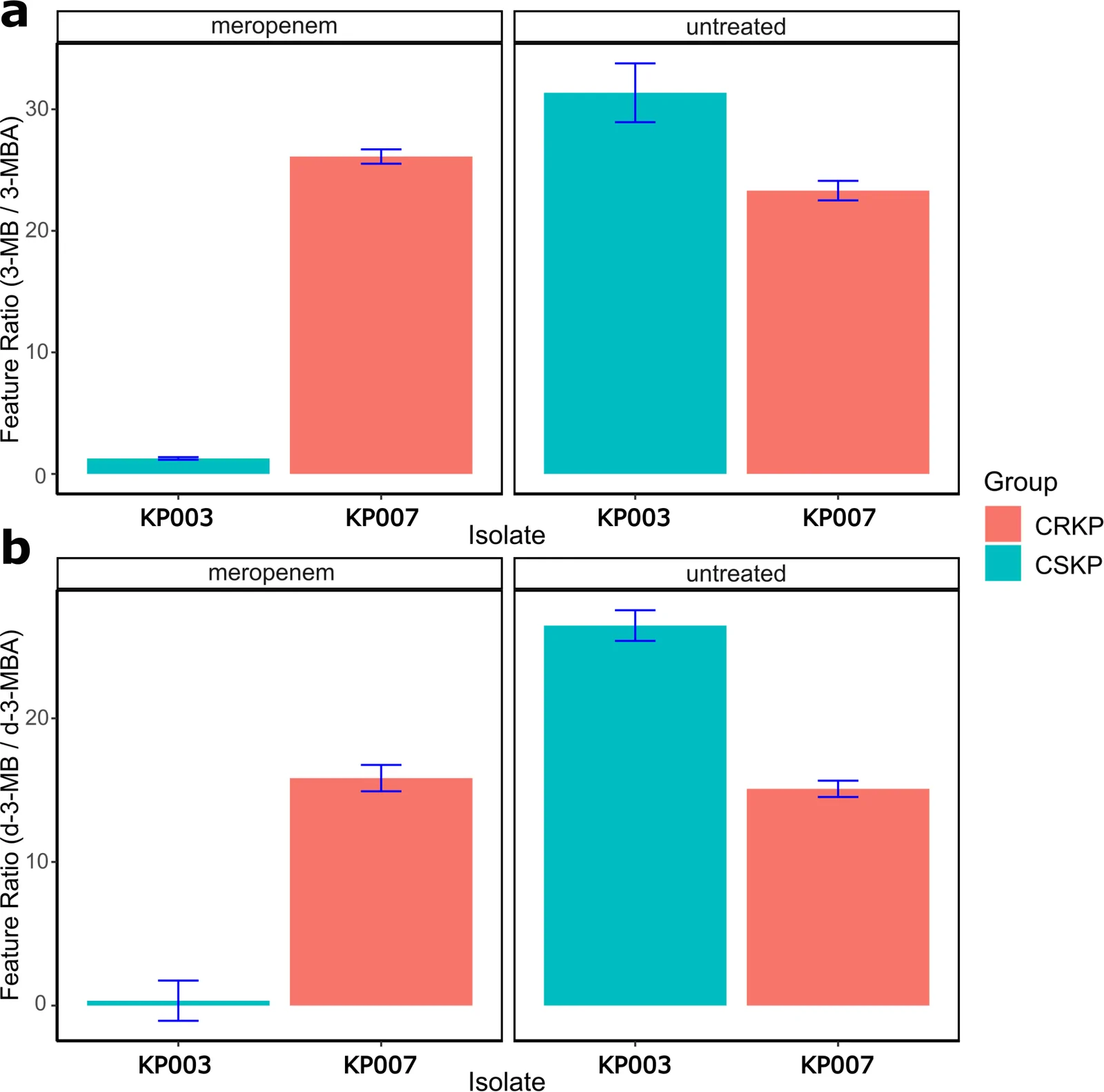
Mean (**a**) 3-methyl-1-butanol-to-3-methylbutanal and (**b**) deuterated 3-methyl-1-butanol-to-3-methylbutanal ratios after growth of *K. pneumoniae* isolates KP003 and KP007 in (**a**) TSB and (**b**) TSB supplemented with d10-leucine both with and without meropenem disks.

To confirm the involvement of the leucine degradation pathway in the generation of the compounds 3-methylbutanal and 3-methyl-1-butanol, we exposed two isolates (KP003 [CSKP] and KP007 [CRKP]) to TSB supplemented with d10-leucine. After growth in the presence of d10-leucine, two distinct peaks were identified eluting immediately prior to the non-deuterated 3-methylbutanal and 3-methyl-1-butanol peaks (Fig. S1). These were confirmed to have mass spectral shifts corresponding to that expected from the use of d10-leucine, validating the involvement of the leucine degradation pathway (Fig. S2 and S3). Furthermore, the deuterated 3-methyl-1-butanol/3-methylbutanal ratios demonstrated congruence with the corresponding non-deuterated compound ratios when meropenem disks were added to the media, suggesting similar pathway utilization and serving as further validation of pathway involvement (Fig. 5a and b).

### Specificity of ratio for *K. pneumoniae* susceptibility

**Fig 6:**
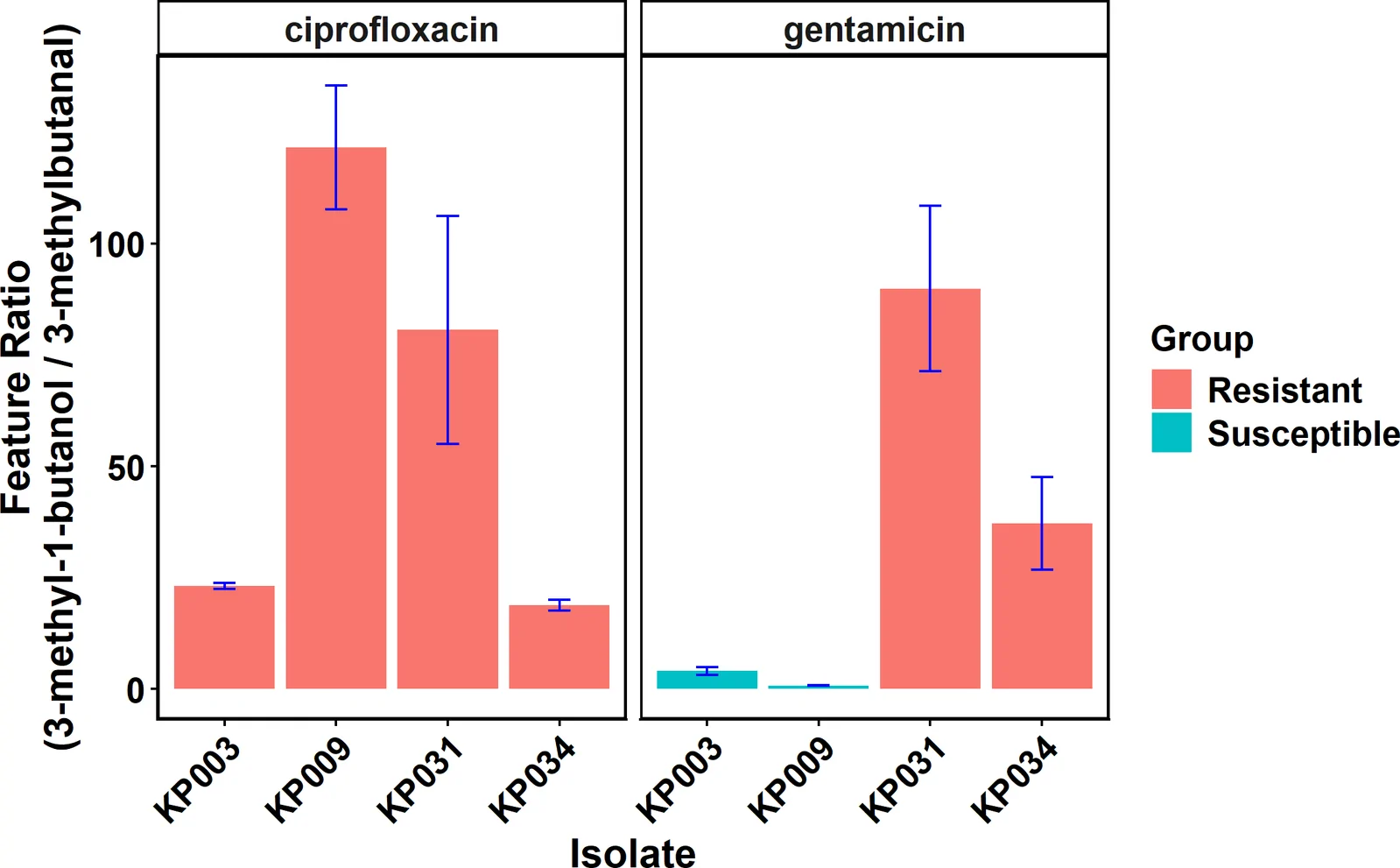
Mean 3-methyl-1-butanol-to-3-methylbutanal ratios of *K. pneumoniae* isolates KP003, KP009, KP031, and KP034 subjected to 5 µg ciprofloxacin or 10 µg gentamicin disks.

To evaluate the specificity of the ratio in characterizing susceptibility, we exposed multiple isolates to ciprofloxacin and gentamicin disks and measured the 3-methyl-1-butanol to 3-methylbutanal ratio. Similar trends were observed with these antibiotics, with a positive correlation between ratio and resistance level, consistent with meropenem (Fig. 6). However, the cut-off threshold of 1.00 did not hold universally as isolate KP003 exhibited a mean ratio of 4.09 (SE = 0.09) despite being susceptible to gentamicin. This suggests that the quantitative metric is specific to meropenem susceptibility.

## DISCUSSION

We have demonstrated that CRKP exhibit a distinct VOC profile, which can be exploited for the detection of meropenem resistance. By using headspace VOC profiling in conjunction with meropenem antibiotic disks, we were able to circumvent the limitations associated with conventional diagnostic methods that typically take days to produce results.

The differential VOC profiles observed between CRKP and CSKP, especially under antibiotic pressure, highlight the utility of microbial metabolites as biomarkers for rapid resistance detection. We assessed the change in VOC levels upon the addition of a meropenem disk and then examined those VOC changes that significantly differed between CSKP and CRKP strains. Our findings revealed that under meropenem stress, benzaldehyde and 3-methylbutanal were elevated in CSKP, while eight other VOCs were higher in CRKP strains. Furthermore, an inverse relationship was observed between the levels of the compounds 3-methyl-1-butanol and 3-methylbutanal.

We have demonstrated that 3-methyl-1-butanol supplementation promotes growth and provides significant protection against meropenem in both susceptible and resistant isolates of *K. pneumoniae.* The protective effect of 3-methyl-1-butanol was more pronounced in the susceptible isolate. We propose that at lower concentrations, 3-methylbutanal is converted to 3-methyl-1-butanol, which may promote growth and confer protection. At higher concentrations, however, this conversion is likely limited, resulting in the accumulation of 3-methylbutanal and associated growth inhibition. This aligns with our observation that susceptible *K. pneumoniae*, when treated with meropenem, accumulates high levels of 3-methylbutanal and exhibits impaired growth.

The observed effects of 3-methyl-1-butanol on bacterial response to meropenem may arise through multiple mechanisms. Alcohols influence the NAD+/NADH ratio, and a more favorable redox balance could accelerate the transition from lag to log phase by enhancing metabolic readiness (34). Furthermore, the reduction of 3-methylbutanal to 3-methyl-1-butanol and thus regeneration of NAD+ is associated with reduced ROS (35). In rice, 3-methyl-1-butanol was found to induce activities in ROS scavengers, augmenting tolerance to oxidative damage (36). Under antibiotic stress, bacteria typically pause growth to synthesize protective proteins during activation of the stress response (37, 38). It is possible that 3-methyl-1-butanol may mitigate stress effects, shortening adaptation time. Last, 3-methyl-1-butanol has demonstrated activity against *in vitro* quorum-sensing systems, suggesting a role for it as a quorum-sensing molecule. In this regard, it may modulate bacterial communication and signal favorable conditions for growth, thereby facilitating a faster transition out of lag phase (39).

Previous studies into the VOC profile of CRKP have reported shared metabolites with those seen in our analysis. Luo et al. recently proposed 3-methyl-1-butanol as a potential VOC biomarker for the identification of KPC- and NDM-producing *K. pneumoniae* upon the addition of imipenem (40). Using gas chromatography-ion mobility spectrometry, Li et al. observed higher levels of 3-methyl-1-butanol, 1-hexanol, and acetoin in CRKP (41). Similarly, benzaldehyde and 3-methylbutanal levels have been shown to be higher in carbapenem-susceptible isolates (27, 41, 42). On the contrary, Filipiak et al. found 3-methyl-1-butanol to be the highest in CSKP in untreated conditions and did not observe any differential change upon imipenem addition (43). Last, avibactam sodium, pyridine-2,6-dicarboxylic acid, and EDTA have all been demonstrated to alter the volatile profile of class A and B carbapenemase producers, permitting the discrimination of these subclasses (40–42).

While 3-methyl-1-butanol has been proposed as a potential biomarker, this is the first study to propose a plausible framework to utilize it for the detection of CRKP. Previously, the distinction between susceptible and resistant isolates was performed using the differences in peak intensity of a single compound to distinguish between groups (40, 41). In our approach, we used ratios to evaluate sample components. The use of ratios provides a semi-quantitative measure without requiring a fully targeted quantitative assay. By doing so, the need for extensive calibration standards is eliminated, and variations in sample preparation, instrument sensitivity, and experimental conditions are likely to have less confounding influence. The robustness of this method allows for more streamlined, cost-effective analysis while still providing meaningful data. Notably, the use of the 3-methyl-1-butanol/3-methylbutanal ratio as a biomarker showed remarkable sensitivity in differentiating CRKP from CSKP. This ratio not only correlated strongly with MIC values but also showed a strong negative correlation with the ZOI, which closely mirrored the correlation between MIC and ZOI. These results suggest that the ratio of these specific metabolites can serve as a reliable measure for assessing carbapenem susceptibility in *K. pneumoniae*.

Our results also demonstrate the robustness of this approach across varying concentrations of meropenem. The ability to distinguish between isolates with different MIC values using the 3-methyl-1-butanol/3-methylbutanal ratio suggests the potential of this method to characterize different levels of susceptibility and provide a quantitative MIC result. One of the key advantages of our approach is the reduction in detection time, with spectral analysis performed after just 6 h of incubation, as opposed to the extended culture periods typically required for susceptibility testing. This reduced turnaround time could be transformative in clinical settings, enabling more timely and targeted antimicrobial therapy, which is critical for improving patient outcomes and reducing the spread of resistance.

Despite the promising results, there are limitations to our study. The sample size of 16 isolates, while sufficient to demonstrate proof-of-concept, is relatively small. Larger-scale studies are needed to validate the clinical applicability of our method across diverse *K. pneumoniae* strains and resistance mechanisms.

Additionally, while TD-GC-MS offers high sensitivity and specificity for VOC analysis, its adoption in routine clinical diagnostics may be constrained by the cost and limited availability of the required instrumentation. Real-time VOC tracer studies may provide additional insight into metabolic flux compared to sorbent-based passive sampling. Future research should therefore focus on developing portable, cost-effective online VOC analysis platforms to facilitate more accessible point-of-care testing. Notably, the compound 3-methyl-1-butanol has been detected in the breath of patients with bacterial lung infections, demonstrating the potential for real-time, non-invasive VOC detection in clinical settings, supported by previous studies and emerging colorimetric platforms designed for the detection of microbial VOCs in clinical laboratories (44–46).

### Conclusion

In conclusion, our study highlights the potential of VOC-based diagnostics as a tool for the faster detection of carbapenem resistance in *K. pneumoniae*, and we propose a new mechanism in which to do so. The identification of specific VOC biomarkers and their correlation with phenotypic resistance profiles offers a novel approach to antimicrobial susceptibility testing, with implications for infection control and antimicrobial stewardship. Further research is needed to translate these findings into practical diagnostic solutions that can be deployed in clinical settings.

## Data Availability

Raw spectral data files and study information are available in the MetaboLights database (47) under study ID MTBLS13539.

## SUPPLEMENTAL MATERIAL

Supplemental material spectrum.00099-26-s0001.pdf Supplemental tables and figures.

ASM does not own the copyrights to Supplemental Material that may be linked to, or accessed through, an article. The authors have granted ASM a non-exclusive, world-wide license to publish the Supplemental Material files. Please contact the corresponding author directly for reuse.
