# Supplementary material for: Leucine degradation metabolite ratio as a measure of meropenem susceptibility in carbapenem-resistant Klebsiella pneumoniae
Source: Microbiol Spectr. 2026 Jun 10;14(7):e00099-26. doi: 10.1128/spectrum.00099-26 (PMC13340099; doi:10.1128/spectrum.00099-26)
Supplement: Supplemental material — Supplemental tables and figures. [file spectrum.00099-26-s0001.pdf]

Supplementary

Supp. Table 1. Meropenem susceptibility testing results of bacterial isolates. Breakpoints: zone diameter; S ≥ 22 mm, R < 16 mm, MIC; S ≤ 2 µg mL^{-1}, R > 8 µg mL^{-1}. S = susceptible, I = susceptible, increased exposure, R = resistant.

Isolate	Group	Meropenem MIC (µg mL^{-1})	Meropenem zone of inhibition (mm)
**KP001**	CSKP	0.064 (S)	25 (S)
**KP003**	CSKP	0.032 (S)	28 (S)
**KP006**	CRKP	128 (R)	7 (R)
**KP007**	CRKP	>128 (R)	6 (R)
**KP008**	CRKP	32 (R)	14 (R)
**KP009**	CRKP	128 (R)	8 (R)
**KP012**	CSKP	0.032 (S)	24 (S)
**KP014**	CSKP	0.064 (S)	25 (S)
**KP019**	CSKP	2 (S)	18 (I)
**KP022**	CRKP	64 (R)	7 (R)
**KP023**	CRKP	128 (R)	6 (R)
**KP025**	CSKP	0.032 (S)	37 (S)
**KP029**	CSKP	0.125 (S)	26 (S)
**KP030**	CRKP	16 (R)	13 (R)
**KP031**	CRKP	>128 (R)	6 (R)
**KP034**	CRKP	128 (R)	6 (R)

Supp. Table 2. Antibiotic susceptibility testing results of bacterial isolates. Breakpoints: gentamicin; S ≥ 17 mm, R < 17 mm, ciprofloxacin; S ≥ 25 mm, R < 22 mm. S = susceptible, R = resistant.

	**Zone of inhibition (mm)**	
**Isolate**	**Gentamicin (10 µg)**	**Ciprofloxacin (5 µg)**
**KP003**	19 (S)	6 (R)
**KP009**	19 (S)	6 (R)
**KP031**	6 (R)	6 (R)
**KP034**	6 (R)	6 (R)

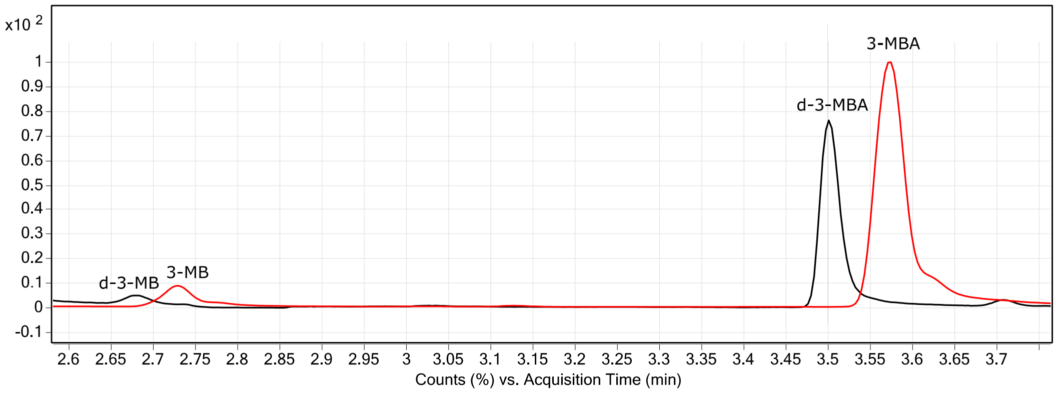

Supp. Figure 1. Extracted ion chromatogram from *K. pneumoniae* headspace when cultured in TSB (red) and d10-leucine supplemented TSB (black), with relevant peaks labelled. TSB trace extracted at *m/z* 44.1 (3-MBA) and 70.1 (3-MB), whilst d10-leucine TSB trace was extracted at *m/z* 47.1 (d-3-MBA) and 60.1 (d-3-MB).

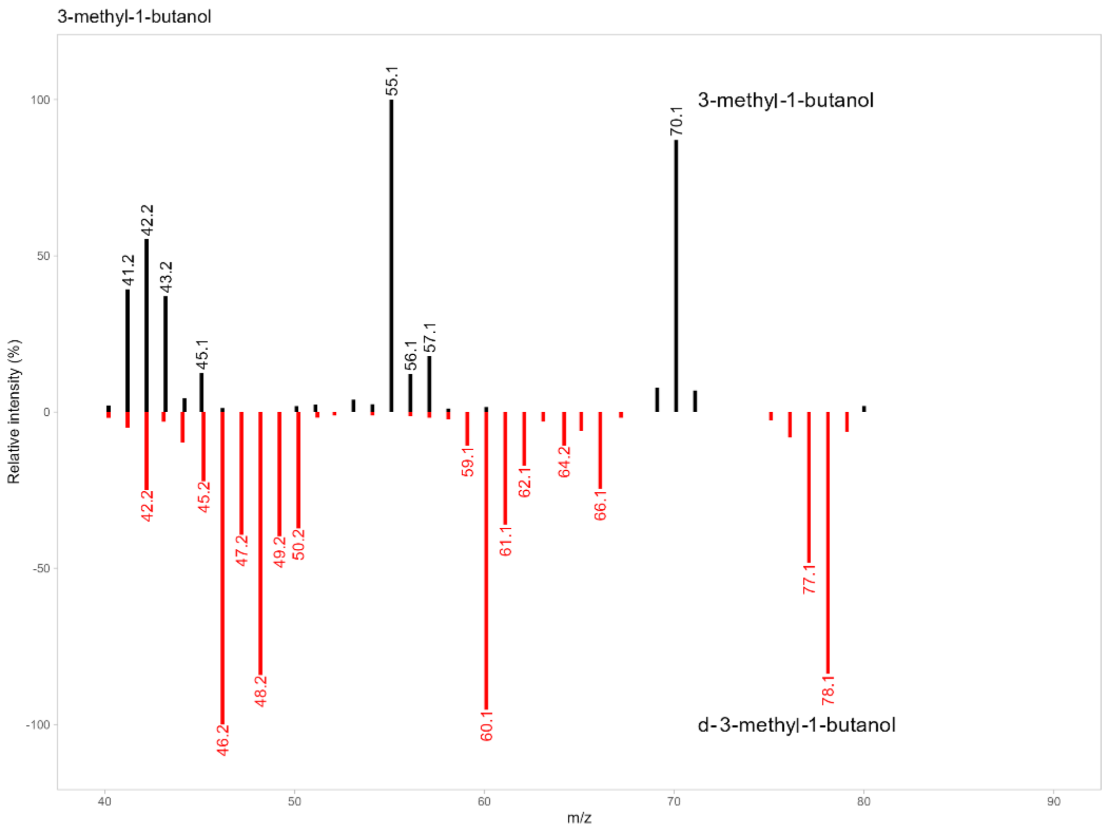

Supp. Figure 2. Mass spectrum mirror plot of 3-methyl-1-butanol when produced with native leucine (top, black) compared with d10-leucine (bottom, red).

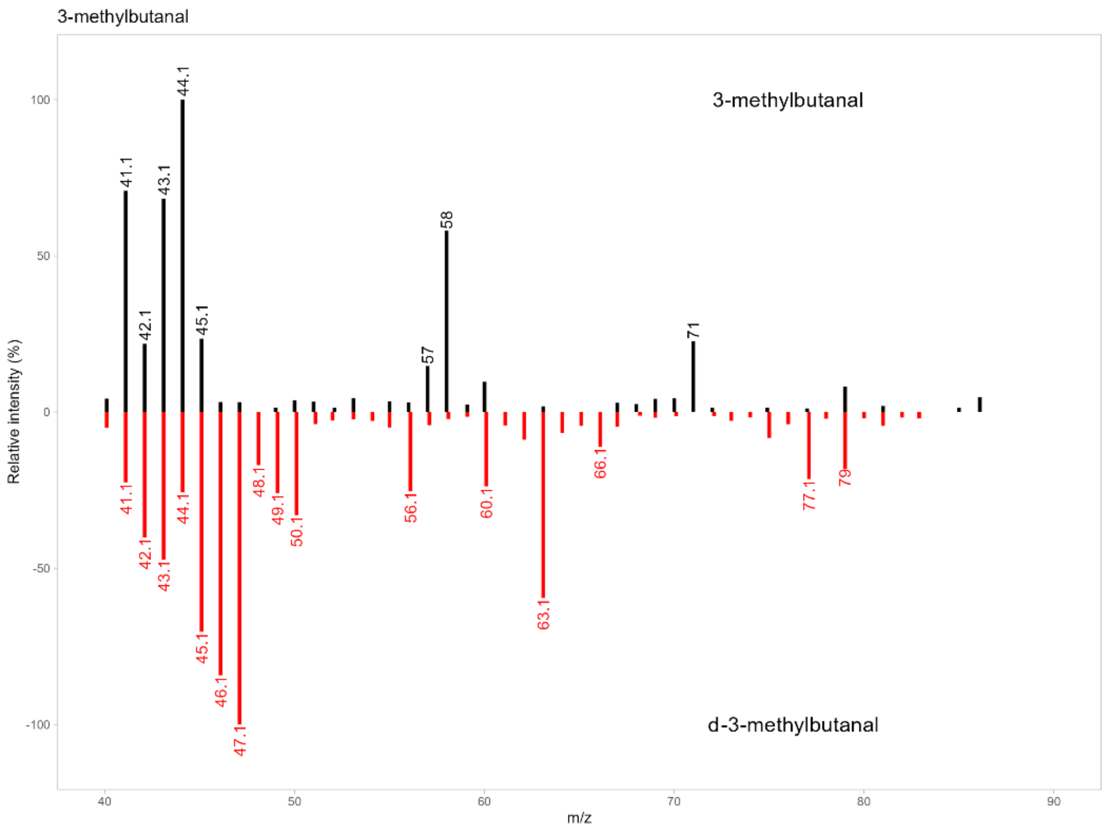

Supp. Figure 3. Mass spectrum mirror plot of 3-methylbutanal when produced with native leucine (top, black) compared with d10-leucine (bottom, red).
